# Sn-doped In_2_O_3_ nanowires: enhancement of electrical field emission by a selective area growth

**DOI:** 10.1186/1556-276X-7-684

**Published:** 2012-12-21

**Authors:** Wen-Chih Chang, Cheng-Hsiang Kuo, Chien-Chang Juan, Pei-Jung Lee, Yu-Lun Chueh, Su-Jien Lin

**Affiliations:** 1 Department of Materials Science and Engineering, National Tsing Hua University, No. 101, Sec. 2, Kuang-Fu Rd., Hsinchu, 30013, Taiwan

**Keywords:** ITO, Nanowire, Field emission, Screen effect.

## Abstract

Selective area growth of single crystalline Sn-doped In_2_O_3_ (ITO) nanowires synthesized via vapor–liquid–solid (VLS) method at 600°C was applied to improve the field emission behavior owing to the reduction of screen effect. The enhanced field emission performance reveals the reduction of turn-on fields from 9.3 to 6.6 V μm^−1^ with increase of field enhancement factors (*β*) from 1,621 to 1,857 after the selective area growth at 3 h. Moreover, we find that the screen effect also highly depends on the length of nanowires on the field emission performance. Consequently, the turn-on fields increase from 6.6 to 13.6 V μm^−1^ with decreasing *β* values from 1,857 to 699 after the 10-h growth. The detailed screen effect in terms of electrical potential and NW density are investigated in details. The findings provide an effective way of improving the field emission properties for nanodevice application.

## Background

Recently, the Sn-doped In_2_O_3_ (indium tin oxide (ITO)) material as a transparent conducting oxides is widely used on many technological applications, such as solar cell [1] and flat panel display [2,3]. Especially in nanoscale region, the Sn-doped In_2_O_3_ (ITO) nanowires have exhibited some superior properties such as good thermal stability, higher metallic conductivity, and excellent oxidation resistance, which make ITO nanowires (NWs) being suitable as a promising candidate not only as a transparent electrode but also as an emitter [4-7]. Up to now, several research groups have reported the growth of ITO nanowires, nanorods, and nanowhisker with different synthetic methods, such as thermal evaporation [8-11], electron beam evaporation [12], sputtering [13], and pulse laser deposition [14]. These nanostructures were found to exhibit a good performance at field emission as an electron emitter due to their high aspect ratio at the nanoscale region and unique extrinsic properties. In the previous report, Wan et al. has reported the epitaxial growth of vertically aligned ITO NWs on the (100) yttrium-stabilized zirconia substrate and showed a superior field emission property [6].

For a good field emission performance from nanowires, it highly depends on the shape of the nanowire [15], circus radius of the nanowire at the tip region [16], work function [17], and packing density of the nanowire [15]. Thus, to obtain the high-density emission sites, one of the most important factors, the screen effect, due to the disturbance of electric field resulting from the interference of emission at different spacings between nanowires must be minimized [18]. Therefore, the selective area growth of nanowires was required. However, how electrical field emission properties of ITO NWs influenced by the screen effect in the differently grown situations is still interesting [19]. Several selective growth methods had been used, such as nanosphere lithography [20], electron-beam lithography [21,22], and conventional photolithography [19].

In this regard, we present a selective area growth of single crystalline Sn-doped ITO NWs to improve the field emission properties owing to the reduction of the screen effect. In our previous study, the conductive properties of ITO NWs have been investigated, which is compatible with that of the high quality ITO thin films [23,24]. A periodically arrayed Au film prepared via a copper grid mask is used to control the growth area of ITO NWs in order to investigate the screen effect. Importantly, the length of ITO NWs was found to significantly influence the field emission properties. As a result, the reduced turn-on fields from 9.3 to 6.6 V μm^−1^ and improved *β* values from 1,621 to 1,857 could be found after the selective area growth of Sn-doped ITO NWs at 3 h.

## Methods

### Growth of Sn-doped ITO nanowires

The ITO nanowires were grown by the hydrogen thermal reduction vapor transport method. Indium (99.9%) and tin (99.9%) were mixed as source powders with the weight ratio of 9:1 and placed in an alumina boat (Al_2_O_3_). The 5-nm-thick Au film as the catalyst was deposited on the silicon substrate by a sputter process and patterned by a copper grid mask. The alumina boat was placed in the center of the alumina tube and then the substrates were put into the low region (several center meters) next to the source powder. The system was heated up to 600°C with a heating rate of 5°C/min. Consequently, the ITO NWs were grown at 600°C for 10 and 3 h with a constant flow of mixed Ar/H_2_ gas (10% H_2_) at 90 sccm. Another oxygen gas was flowed into the furnace with 0.5 sccm as a source of oxygen to form ITO NWs. After the furnace had been cooled down to room temperature, gray products were found on the surface of the silicon substrate.

### Characterization

Structures of products were analyzed by X-ray diffractometer (XRD, Shimadzu XRD 6000, Nakagyo-ku, Kyoto, Japan) and transmission electron microscope (TEM, JEOL-2010, JEOL Ltd., Akishima, Tokyo, Japan). The morphology was analyzed by field emission scanning electron microscope (SEM, JEOL-6500). The X-ray photoelectron spectroscopy (XPS, ULVAC-PHI, PHI Quantera SXM, Chanhassen, MN, USA) was used to examine the chemical composition of nanowires. Field emission measurement of ITO NW arrays was performed with a parallel plate as the cathode and a circular steeliness tip as the anode (1-mm diameter). A high voltage–current instrument, Keithley 237 (Cleveland, OH, USA), was operated to perform the field emission characteristics. All emission measurements were carried out in a vacuum chamber with a pressure kept under 10^−6^ Torr The applied voltage between the electrodes was increased to a maximum of 1,000 V by 20-V step.

## Results and discussion

**Figure 1:**
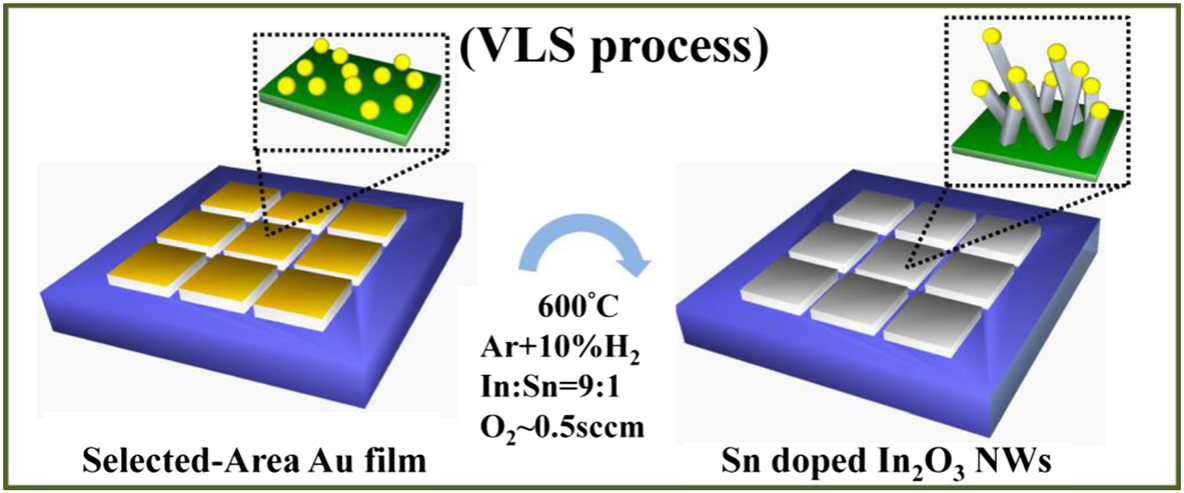
Schematics for the selective area growth of ITO nanowire growth.

Figure 1 shows the growth of ITO NWs catalyzed by a selected-area gold film. According to the vapor–liquid–solid (VLS) growth mechanism [25-27], the possible reaction routes can be assumed as follows:

(1) $$2In_{\left(g\right)}+\frac{1}{2}O_{2\left(g\right)}\to In_{2}O_{\left(g\right)}$$

(2) $$Sn_{\left(g\right)}+\frac{1}{2}O_{2\left(g\right)}\to In_{2}O_{\left(g\right)}$$

(3) $$In_{2}O_{\left(g\right)}+H_{2\left(g\right)}\to 2{\left[\mathrm{In}\right]}_{\left(\mathrm{Au}\right)}+H_{2}O_{\left(g\right)}$$

(4) $$\mathrm{Sn}O_{2\left(g\right)}+2H_{2\left(g\right)}\to {\left[\mathrm{Sn}\right]}_{\left(\mathrm{Au}\right)}+2H_{2}O_{\left(g\right)}$$

(5) $$2{\left[\mathrm{In}\right]}_{\left(\mathrm{Au}\right)}+3H_{2}O_{\left(g\right)}\to In_{2}O_{3\left(s\right)}+3H_{2\left(g\right)}$$

(6) $${\left[\mathrm{Sn}\right]}_{\left(\mathrm{Au}\right)}+2H_{2}O_{\left(g\right)}\to \mathrm{Sn}O_{2\left(s\right)}+2H_{2\left(g\right)}$$

The reaction of the VLS method is at a high-temperature environment. As the temperature increases to 600°C, the Au drops could be formed, and the low melting point of the source powder (In and Sn) is evaporated to combine with oxygen gas to form metal oxide gases (In_2_O_3_, SnO_2_) through the chemical reactions of Equations 1 and 2. Subsequently, the metal oxide gases could be reduced by hydrogen to form the metal atoms and then enter to the liquid gold drops to form eutectic alloy through Equations 3 and 4. Furthermore, hydrogen and oxygen could combine to form H_2_O. Finally, the eutectic alloy drops would be oxidized to form the Sn-doped In_2_O_3_ NWs by H_2_O, namely, Equations 5 and 6. When the temperature increased to 600°C, the oxygen would be introduced into the alumina tube, resulting in the oxidization of In and Sn vapors, with which the growth time would be conducted at 600°C for 3 and 10 h.

**Figure 2:**
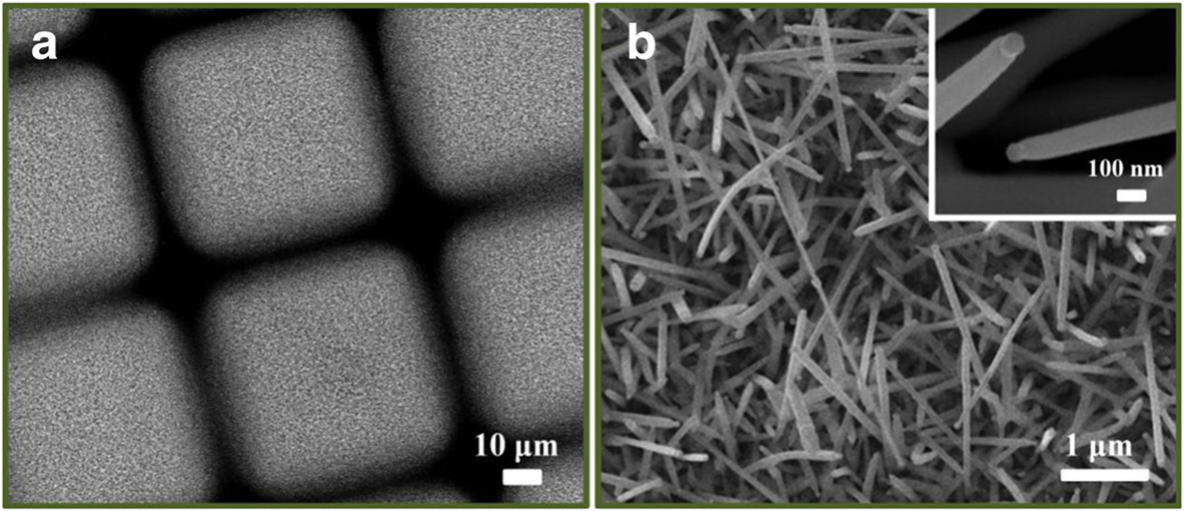
**SEM images.** (**a**) A SEM image of the selective area growth of ITO nanowires. (**b**) Enlarged SEM image taken from the center of the patterned area. The inset shows an ITO nanowire with catalytic gold nanoparticle.

To decrease the screening effect on the arbitrarily grown ITO NWs, the Sn-doped ITO NWs were alternatively grown on the Au film with the selective area of patterned 50-μm square with a distance of 10 μm for each square pattern. Figure 2a reveals a SEM image of Sn-doped ITO nanowires after the selective area growth. Clearly, the center of the patterned area shows the arbitrary growth of ITO NWs (Figure 2b), and the inset shows ITO nanowires with catalytic Au nanoparticles, confirming the VLS method of Sn-doped ITO NWs. In addition, the dispersion of ITO nanowire diameter ranges from 40 to approximately 200 nm with an average diameter of 110 nm.

**Figure 3:**
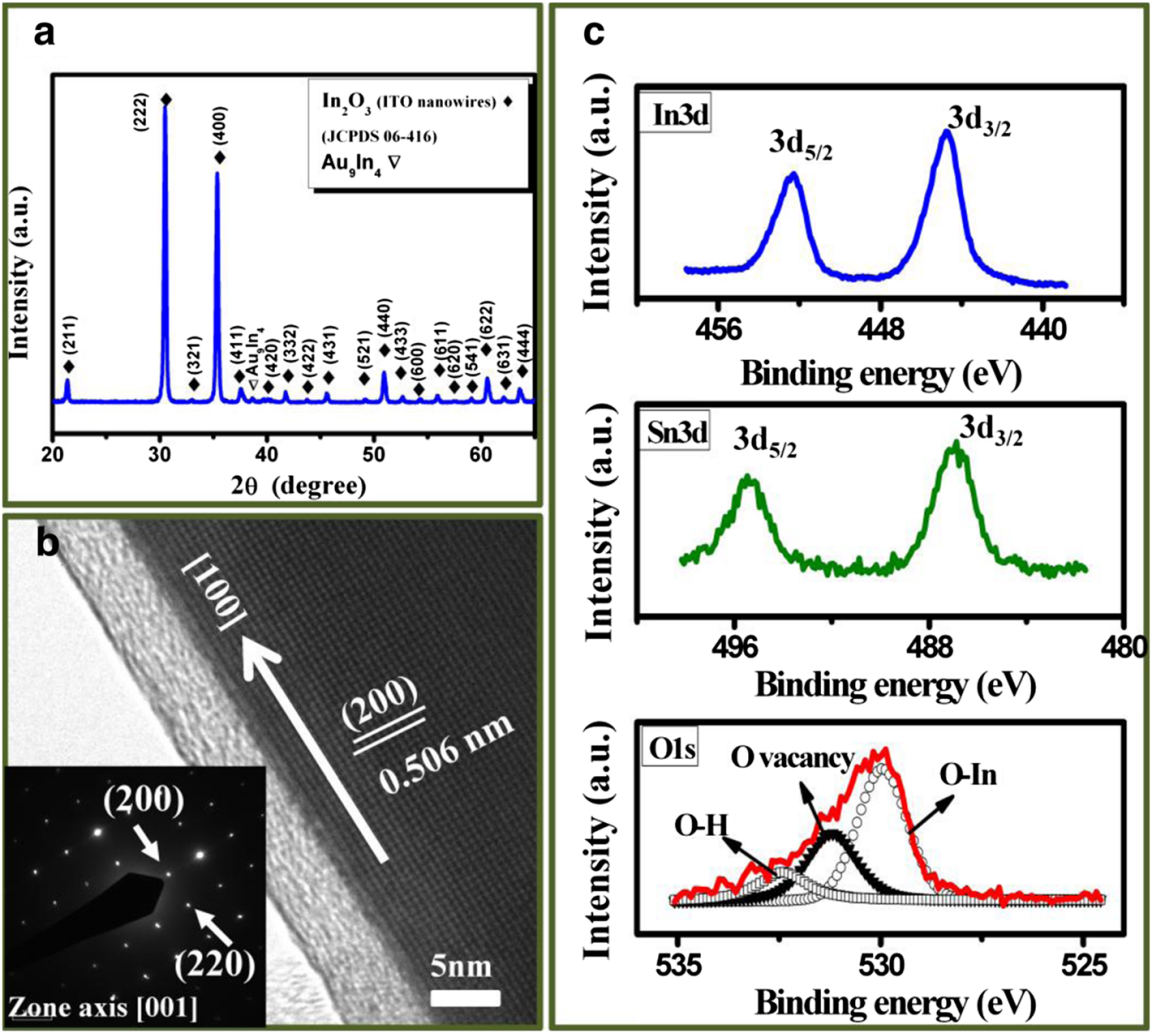
**XRD spectra and high-resolution TEM image.** (**a**) XRD spectra of ITO NWs. (**b**) A high-resolution TEM image of ITO nanowire. The inset shows a corresponding selective area diffraction pattern, revealing that [100] is a preferred growth direction. (**c**) Chemical bonding information of In, Sn, and O for the ITO NWs extracted from the XPS spectra.

To illuminate the detailed structure and components of the ITO NWs, the as-prepared nanowires were characterized by XRD, TEM, and XPS. Figure 3a shows the X-ray spectra of ITO NWs. All the peaks are indexed being the In_2_O_3_ cubic structure, while a small peak shows Au_9_In_4_ phase, which comes from the catalytic gold nanoparticles on the top of ITO nanowires. Furthermore, the high-resolution TEM image and the corresponding selected area electron diffraction (SAED) pattern with zone axis of [001] are shown in Figure 3b and the inset, respectively. The symmetric spots in the SAED pattern exhibit a single crystalline phase with the growth direction of [100]. The lattice spacing of 0.506 nm corresponding to (200) plane was indexed, which is consistent with In_2_O_3_ cubic phase. The XPS analysis is used to confirm the chemical compositions of ITO NWs. Figure 3c shows the XPS spectra of O 1*s*, In 3*d*, and Sn 3*d* core levels in the ITO NWs. The binding energy of Sn 3*d*_5/2_ and Sn 3*d*_3/2_ at 495.1 ± 0.1 eV and 486.6 ± 0.1 eV, correspond to the Sn^4+^ ion, respectively, which are relative to the electrical conduction of the nanowires [28]. The O 1*s* peak is deconvoluted by a Gaussian function into three positions. The lower binding energy component at 530 ± 0.1 eV is due to the O^2−^ ions whose neighboring indium atoms are surrounded by the six nearest O^2−^ ions. The medium binding energy at 531.3 ± 0.1 eV corresponds to the oxygen deficiency regions, which are called oxygen vacancies [28,29]. The higher binding energy at 532.6 ± 0.1 eV is associated with the oxygen of free hydroxyl group, which is possibly due to the water molecules absorbed on the surface [30]. All XPS results show that Sn atoms are doped into the In_2_O_3_ NWs with the existence of oxygen vacancies. Consequently, the oxygen vacancies and Sn ions contribute the electron concentration to the NWs, resulting in an n-type semiconducting behavior.

**Figure 4:**
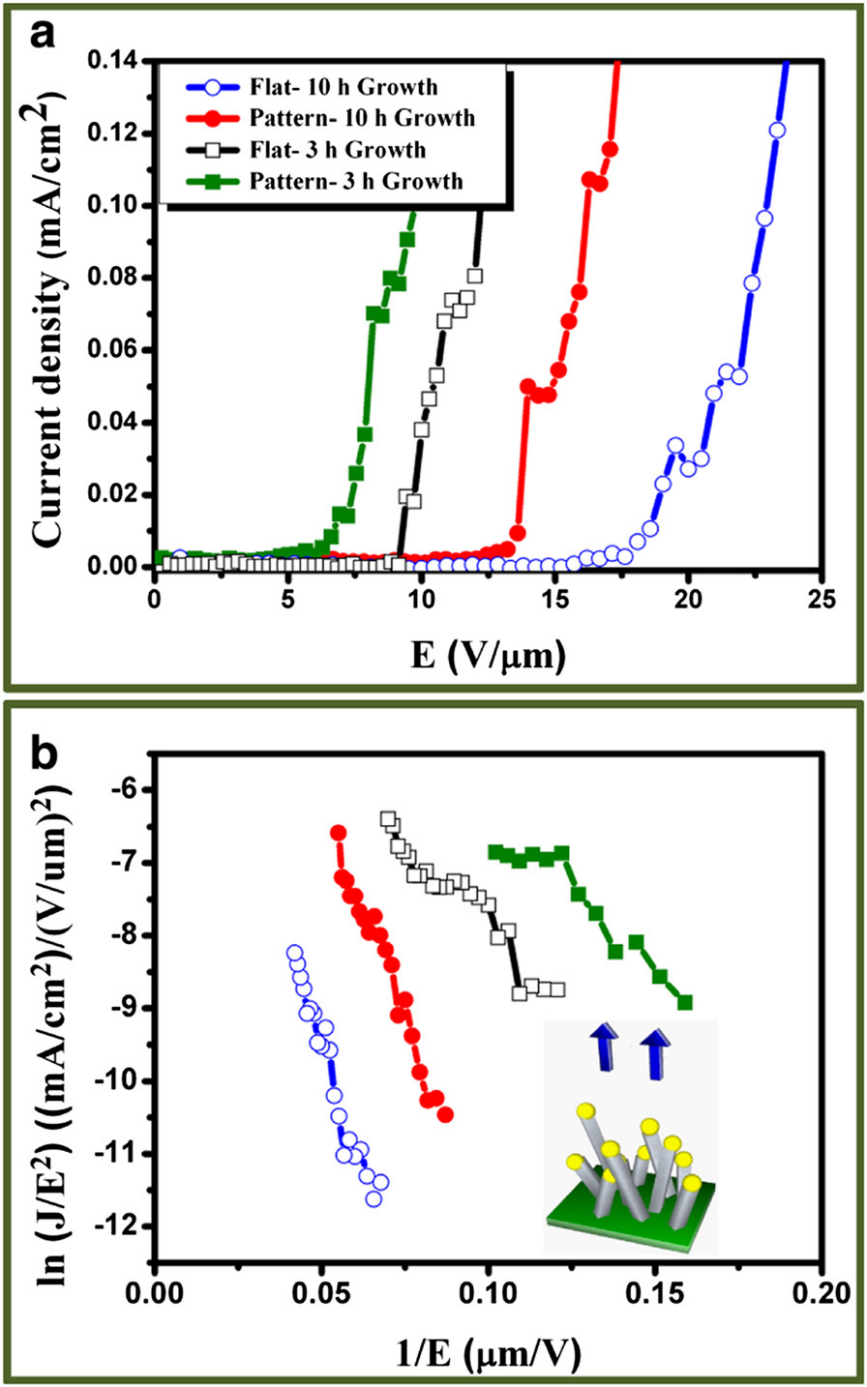
***J*-*E *field emission curves and Fowler-Nordheim plots.** (**a**) *J*-*E* field emission curves for flat and selectively patterned growth at 3 and 10 h, respectively. (**b**) The corresponding Fowler-Nordheim plots from (a) for four samples.

Figure 4a shows field emission properties of the ITO NWs grown on Au film and patterned Au film with growth time of 3 and 10 h, respectively. The turn-on field (*E*_on_) is defined as the electric field required for generating a current density of 0.01 mA/cm^2^, and 0.1 mA/cm^2^ is sufficient for operating display panel devices. It is found that the turn-on field decreases from 9.3 to 6.6 V μm^−1^ after the selective area growth of ITO NWs at the growth time of 3 h. Insets in Figure 4b reveal a linear relationship, so-called ln(*J*/*E*^2^)-(1/*E*) plot, indicating that the field-emission behavior follows Fowler-Nordheim relationship, i.e., electrons tunneling through a potential barrier, which can be expressed as follows [31-33]:

(7) J=Aβ2E2ϕexp−Bϕ32βE,

where *J* is the emission current density; *E*, the applied field; *ϕ*, the work function of emitter material; *β*, the enhancement factor; *A*, constant (1.56 × 10^−10^ A V^−2^ eV); and *B*, constant (6.8 ×10^3^ eV^−3/2^ V μm^−1^) The field enhancement factor, *β*, reflects the degree of the field emission enhancement of the tip shape on a planar surface, which is also dependent on the geometry of the nanowire, the crystal structure, and the density at the emitting points. It can be determined by the slope of the ln(*J*/*E*^2^)-(1/*E*) plot with a work function value of 4.3 eV [6]. Consequently, the turn-on fields and the *β* values of the ITO NWs with and without selective area growth at different growth times are listed in Table 1. Obviously, the field enhancement factors (*β*) from 1,621 to 1,857 can be achieved after the selective area growth at 3 h. Moreover, we find that the screen effect also highly depends on the length of nanowires on the field emission performance. As a result, the turn-on fields increase from 6.6 to 13.6 V μm^−1^, and *β* values decrease from 1,857 to 699 after 10-h growth. Compared to the *β* values of other materials, such as Si nanowires (*β* = 1,000) [34], NiSi_2_ nanorods (*β* = 630) [35], NiSi_2_ nanowires (*β* = 501) [36], SnO_2_ (*β* = 1402.9) [37], AlN (*β* = 950) [38], and ZnO (*β* = 1,464) [39], the Sn-doped ITO NWs are promising emitters. The findings indicate that the less stacking density via the selective area growth and the reduction of the NW length could decrease the screen effect, resulting in the increase of the enhancement factor.

**Table 1:** Turn-on fields and field enhancement factors for the growth of the ITO NWs at different conditions

	***E***_**on**_**(V μm**^**−1**^**) at *J *= 0.01 mA cm**^**−2**^	***β***
Flat 10-h growth	18	429
Patterned 10-h growth	13.6	699
Flat 3-h growth	9.3	1,621
Patterned 3-h growth	6.6	1,857

**Figure 5:**
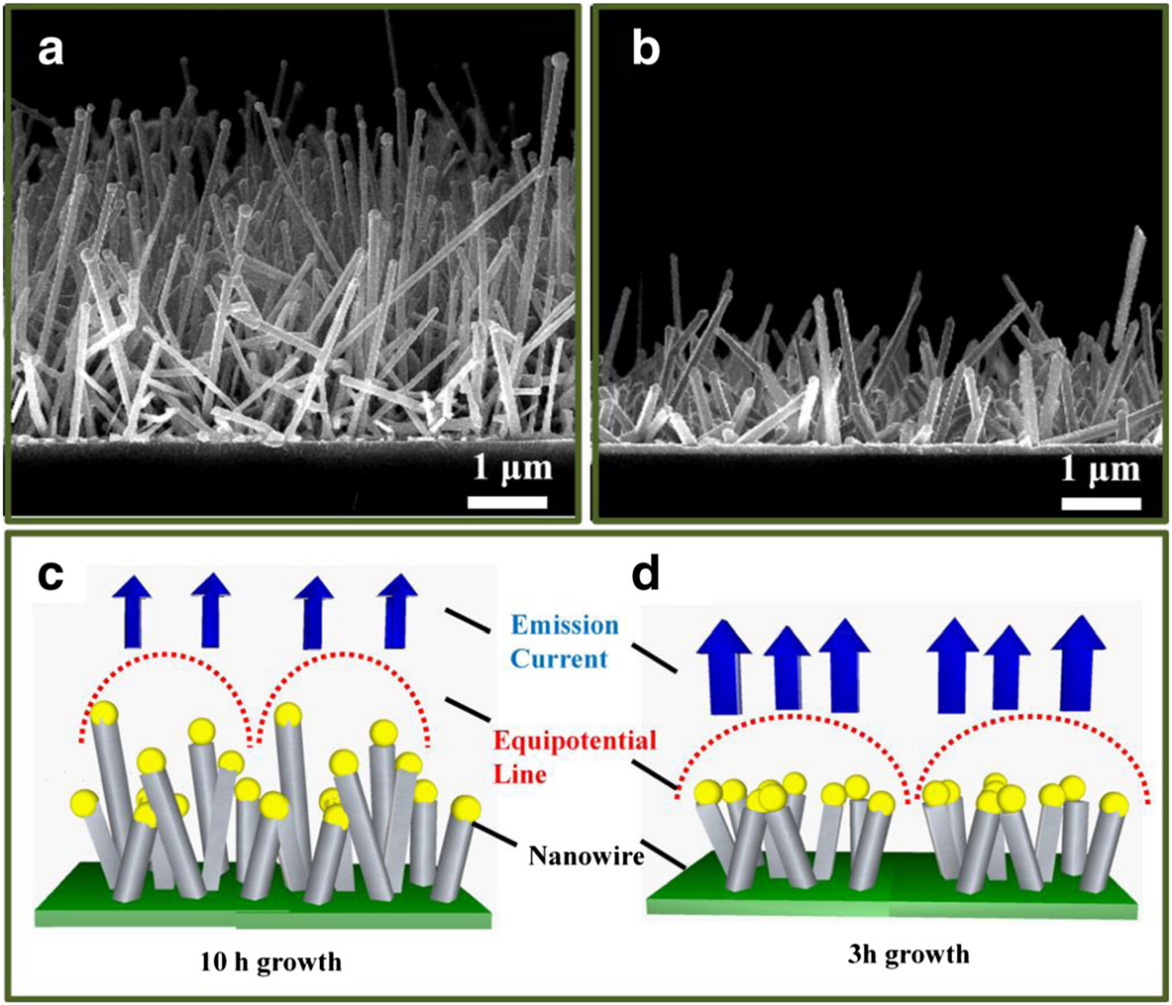
**Cross-sectional SEM images for ITO NWs.** NWs grown at (**a**) 10 and (**b**) 3 h, respectively. (**c**) and (**d**) The corresponding distribution of emission current and electric potential for ITO NWs grown at10 and 3 h, respectively.

The cross-sectional SEM images for the growth of Sn-doped ITO NWs at 10 and 3 h are shown in Figure 5a,b to confirm the reduction of the screen effect, respectively. Obviously, ITO NWs are tangled together due to the longer length (10-h growth), while the quasi-vertical growth could be achieved at the shorter time (3-h growth). According to the screening effect, the electrical field around ITO NWs with longer length and random growth would interfere together to result in screen effect, thereby a poor field emission [40,41]. The corresponding potential distribution of the ITO NWs for Sn-doped ITO NWs grown at 10 and 3 h related to the electrical field are shown in Figure 5c,d, respectively. Notably, Figure 5c (10-h growth) reveals that the NWs significantly tangled together, resulting in lower current emission because of the lesser equipotential lines owing to the server screen effect. Therefore, only the higher NWs would emit current. On the contrary, Figure 5d (3-h growth) reveals that the shorter NWs could decrease the screen effect due to the much larger dispersive equipotential lines around the NWs, triggering a higher current emission. This is why the shorter grown time of ITO NWs shows the much better FE property. The findings provide an effective way of improving the field emission properties for nanodevice application.

## Conclusion

We present a selective area growth of single crystalline Sn-doped In_2_O_3_ (ITO) nanowires synthesized via VLS method at 600°C in order to improve the field emission behavior by the reduction of screen effect. The enhanced field emission performance reveals the reduction of turn-on fields from 9.3 to 6.6 V μm^−1^ with increase of field enhancement factors (*β*) from 1,621 to 1857 after the selective area growth at 3 h. Moreover, we find that the screen effect also highly depends on the length of nanowires on the field emission performance. The turn-on fields increase from 6.6 to 13.6 V μm^−1^, and *β* values decrease from 1,857 to 699 after the 10-h growth. The screen effect is predominated after the length of nanowires increases, namely the longer growth time, thereby degrading the field emission performance. Consequently, the turn-on fields and *β* values change from 13.6 V μm^−1^ and 699 to 6.6 V μm^−1^ and 1,857, respectively, as the growth time of Sn-doped ITO NWs decreases into 3 h. The detailed screen effect in terms of electrical potential and NW density was investigated in details. The findings provide an effective way of improving the field emission properties for nano-emitter application.

## Competing interests

The authors declare that they have no competing interests.

## Authors’ contributions

WCC operated the SEM instrument and measured the FE property. PJL deposited the gold film of Si sample. CCJ operated the TEM instrument. CHK carried out the XPS characterization. SJL and YLC support the information and organized the final version of the paper. All authors read and approved the final manuscript.

## References

[B1] Ngamsinlapasathian S Sreethawong T Suzuki Y Yoshikawa S Doubled layered ITO/SnO_2_ conducting glass for substrate of dye-sensitized solar cells Sol Energy Mater Sol Cells 2006 90 2129 2140 10.1016/j.solmat.2005.12.005

[B2] Kamei M Yagami T Takaki S Shigesato Y Heteroepitaxial growth of tin-doped indium oxide films on single crystalline yttria stabilized zirconia substrates Appl Phys Lett 1994 64 2712 2714 10.1063/1.111474

[B3] Ohta H Orita M Hirano M Tanji H Kawazoe H Hosono H Highly electrically conductive indium–tin–oxide thin films epitaxially grown on yttria-stabilized zirconia (100) by pulsed-laser deposition Appl Phys Lett 2000 76 2740 10.1063/1.126461

[B4] O’Dwyer C Szachowicz M Visimberga G Lavayen V Newcomb S Torres C Bottom-up growth of fully transparent contact layers of indium tin oxide nanowires for light-emitting devices Nat Nanotechnol 2009 4 239 244 10.1038/nnano.2008.418 19350034

[B5] Gao J Chen R Li DH Jiang L Ye JC Ma XC Chen XD Xiong QH Sun HD Wu T UV light emitting transparent conducting tin-doped indium oxide (ITO) nanowires Nanotechnol 2011 22 195706 10.1088/0957-4484/22/19/195706 21430316

[B6] Wan Q Feng P Wang TH Vertically aligned tin-doped indium oxide nanowire arrays: epitaxial growth and electron field emission properties Appl Phys Lett 2006 89 123102 10.1063/1.2345278

[B7] Wan Q Dattoli E Fung W Guo W Chen Y Pan X Lu W High-performance transparent conducting oxide nanowires Nano Lett 2006 6 2909 2915 10.1021/nl062213d 17163729

[B8] Peng XS Meng GW Wang XF Wang YW Zhang J Liu X Zhang LD Synthesis of oxygen-deficient indium-tin-oxide (ITO) nanofibers Chem Mater 2002 14 4490 4493 10.1021/cm025567o

[B9] Lee SY Lee CY Lin P Tseng TY Low temperature synthesized Sn doped indium oxide nanowires Nanotechnol 2005 16 451 457 10.1088/0957-4484/16/4/021

[B10] Orlandi MO Aguiar R Lanfredi AJC Longo E Varela JA Leite ER Tin-doped indium oxide nanobelts grown by carbothermal reduction method Appl Phys A: Mater Sci Process 2005 80 23 25 10.1007/s00339-004-3027-x

[B11] Wan Q Wei M Zhi D MacManus-Driscoll JL Blamire MG Epitaxial growth of vertically aligned and branched single‐crystalline tin‐doped indium oxide nanowire arrays Adv Mater 2006 18 234 238 10.1002/adma.200501673

[B12] Pokaipisit A Udomkan N Limsuwan P Nanostructure and properties of indium tin oxide (ITO) films produced by electron beam evaporation Mod Phys Lett B 2006 20 1049 1058 10.1142/S0217984906011402

[B13] Fung MK Sun YC Ng AMC Chen XY Wong KK Djuriši’ c AB Chan WK Indium tin oxide nanowires growth by dc sputtering Appl Phys A 2011 104 1075 1080 10.1007/s00339-011-6372-6

[B14] Yong TK Tan SS Nee CH Yap SS Kee YY György S Zsolt Endre H Jason M Yoke-Khin Y Teck-Yong T Pulsed laser deposition of indium tin oxide nanowires in argon and helium Mater Lett 2012 66 280 281 10.1016/j.matlet.2011.08.085

[B15] Wu JM Characterizing and comparing the cathodoluminesence and field emission properties of Sb doped SnO_2_ and SnO_2_ nanowires Thin Solid Films 2009 517 1289 1293

[B16] Chen LH Hong KH Xiao DQ Hsieh WJ Lai SH Lin TC Shieu FS Chen KJ Cheng HC Role of extrinsic atoms on the morphology and field emission properties of carbon nanotubes Appl Phys Lett 2003 82 4334 10.1063/1.1579136

[B17] Fang CW Wu JM Lee LT Hsien YH Lo SC Chen CH ZnO:Al nanostructures synthesized on pre-deposited aluminum (Al)/Si template: formation, photoluminescence and electron field emission Thin Solid Films 2008 517 1268 1273 10.1016/j.tsf.2008.06.037

[B18] Bonard JM Weiss N Kind H Stockli T Forro L Kern K Chatelain A Tuning the field emission properties of patterned carbon nanotube films Adv Mater 2001 13 184 10.1002/1521-4095(200102)13:3<184::AID-ADMA184>3.0.CO;2-I

[B19] Liu N Fang G Zeng W Long H Yuan L Zhao X Diminish the screen effect in field emission via patterned and selective edge growth of ZnO nanorod arrays Appl Phys Lett 2009 95 153505 10.1063/1.3247887

[B20] Fan HJ Fuhrmann B Scholz R Syrowatka F Dadgar A Krost A Zacharias M Well-ordered ZnO nanowire arrays on GaN substrate fabricated via nanosphere lithography J Cryst Growth 2006 287 34 38 10.1016/j.jcrysgro.2005.10.038

[B21] Kim YJ Yoo J Kwon BH Hong YJ Lee CH Yi GC Position-controlled ZnO nanoflower arrays grown on glass substrates for electron emitter application Nanotechnol 2008 19 315202 10.1088/0957-4484/19/31/315202 21828781

[B22] Ahsanulhaq Q Kim JH Hahn YB Controlled selective growth of ZnO nanorod arrays and their field emission properties Nanotechnol 2007 18 485307 10.1088/0957-4484/18/48/485307

[B23] Nishio K Sei T Tsuchiya T Dip-coating of ITO films J Mater Sci 1996 31 1761 1766 10.1007/BF00372189

[B24] Chang WC Kuo CH Lee PJ Chueh YL Lin SJ Synthesis of single crystal Sn-doped In2O3 nanowires: size-dependent conductive characteristic Phys Chem Chem Phys 2012 14 13041 13045 22886004 10.1039/c2cp41671a

[B25] Wagner RS Ellis WC Vapor‐liquid‐solid mechanism of single crystal growth Appl Phys Lett 1964 4 89 90 10.1063/1.1753975

[B26] Valderrama J Jacob KT Vapor pressure and dissociation energy of (In_2_O) Thermochim Acta 1977 21 215 224 10.1016/0040-6031(77)85019-3

[B27] Liang C Meng G Lei Y Phillipp F Zhang L Catalytic growth of semiconducting In_2_O_3_ nanofibers Adv Mater 2001 13 1330 10.1002/1521-4095(200109)13:17<1330::AID-ADMA1330>3.0.CO;2-6

[B28] Fan JCC Goodenough JB X-ray photoemission spectroscopy studies of Sn-doped indium-oxide films J Appl Phys 1977 48 3524 3531 10.1063/1.324149

[B29] Wu WF Chiou BS Effect of oxygen concentration in the sputtering ambient on the microstructure, electrical and optical properties of radio-frequency magnetron-sputtered indium tin oxide films Semicond Part Sci Technol 1996 11 196 202 10.1088/0268-1242/11/2/009

[B30] Carvalho CN Rego AMB Amaral A Brogueira P Lavareda G Effect of substrate temperature on the surface structure, composition and morphology of indium-tin oxide films Surf CoatTechnol 2000 124 70 75 10.1016/S0257-8972(99)00619-2

[B31] Fowler RH Nordheim L Electron emission in intense electric fields Proc R Soc London, Ser A 1928 119 173 181 10.1098/rspa.1928.0091

[B32] Edgcombe CJ Valdre U Experimental and computational study of field emission characteristics from amorphous carbon single nanotips grown by carbon contamination - I Experiments and computation. Philos Mag B 2002 82 987

[B33] Filip V Nicolaescu D Tanemura M Okuyama F Modeling the electron field emission from carbon nanotube films Ultramicroscopy 2001 89 39 49 10.1016/S0304-3991(01)00107-3 11770750

[B34] Chueh YL Chou LJ Cheng SL He JH We WW Chen LJ Synthesis of taperlike Si nanowires with strong field emission Appl Phys Lett 2005 86 133112 10.1063/1.1883316

[B35] Ok YW Seong TY Choi CJ Tu KN Field emission from Ni-disilicide nanorods formed by using implantation of Ni in Si coupled with laser annealing Appl Phys Lett 2006 88 043106 10.1063/1.2167797

[B36] Lee KS Mo YH Nahm KS Shim HW Suh EK Kim JR Kim JJ Anomalous growth and characterization of carbon-coated nickel silicide nanowires Chem Phys Lett 2004 384 215 10.1016/j.cplett.2003.11.107

[B37] He JH Wu TH Hsin CL Li KM Chen LJ Chueh YL Chou LJ Wang ZL Beaklike SnO2 nanorods with strong photoluminescent and field-emission properties Small 2006 2 116 10.1002/smll.200500210 17193566

[B38] Zhu W Kochanski GP Jin S Seibles L Jacobson D McCormack CM White AE Electron field emission from ion implanted diamond Appl Phys Lett 1995 67 1157 10.1063/1.114993

[B39] Tseng YK Huang CJ Cheng HM Kin IN Liu KS Chen IC Characterization and field-emission properties of needle-like zinc oxide nanowires grown vertically on conductive zinc oxide films Adv Funct Mater 2003 87 73109

[B40] Li SY Lin P Lee CY Tseng TY Field emission and photo fluorescence characteristics of zinc oxide nanowires synthesized by a metal catalyzed vapor–liquid–solid process J Appl Phys 2004 95 3711 3716 10.1063/1.1655685

[B41] Chen ZH Tang YB Liu Y Yuan GD Zhang WF Zapien JA Belloa I Zhang WJ Lee CS Lee ST ZnO nanowire arrays grown on Al:ZnO buffer layers and their enhanced electron field emission J Appl Phys 2009 106 064303 10.1063/1.3213091

